# Quantitative DNA Methylation Analysis and Epigenotype-Phenotype Correlations in Taiwanese Patients with Silver-Russell Syndrome

**DOI:** 10.7150/ijms.84154

**Published:** 2024-01-01

**Authors:** Hsiang-Yu Lin, Chung-Lin Lee, Yuan-Rong Tu, Ya-Hui Chang, Dau-Ming Niu, Chia-Ying Chang, Pao Chin Chiu, Yen-Yin Chou, Hui-Pin Hsiao, Meng-Che Tsai, Mei-Chyn Chao, Li-Ping Tsai, Chia-Feng Yang, Pen-Hua Su, Yu-Wen Pan, Chen-Hao Lee, Tzu-Hung Chu, Chih-Kuang Chuang, Shuan-Pei Lin

**Affiliations:** 1 Department of Medicine, MacKay Medical College, New Taipei City, Taiwan.; 2 Department of Pediatrics, MacKay Memorial Hospital, Taipei, Taiwan.; 3 Department of Medical Research, MacKay Memorial Hospital, Taipei, Taiwan.; 4 MacKay Junior College of Medicine, Nursing and Management, Taipei, Taiwan.; 5 Department of Medical Research, China Medical University Hospital, China Medical University, Taichung, Taiwan.; 6 Department of Rare Disease Center, MacKay Memorial Hospital, Taipei, Taiwan.; 7 Institute of Clinical Medicine, National Yang-Ming University, Taipei, Taiwan.; 8 Institute of Clinical Medicine, National Yang-Ming Chiao Tung University, Taipei, Taiwan.; 9 Department of Pediatrics, Taipei Veterans General Hospital, Taipei, Taiwan.; 10 Department of Pediatrics, MacKay Memorial Hospital, Hsinchu, Taiwan.; 11 Department of Pediatrics, Kaohsiung Veterans General Hospital, Kaohsiung, Taiwan.; 12 Department of Pediatrics, National Cheng Kung University Hospital, Tainan, Taiwan.; 13 Department of Pediatrics, Kaohsiung Medical University Chung Ho Memorial Hospital, Kaohsiung, Taiwan.; 14 Department of Pediatrics, Changhua Christian Children's Hospital, Changhua, Taiwan.; 15 Department of Pediatrics, Taipei Tzu Chi Hospital, Buddhist Tzu Chi Medical Foundation, New Taipei City, Taiwan.; 16 Department of Pediatrics, Chung Shan Medical University, Taichung, Taiwan.; 17 Department of Pediatrics, Chang Gung Memorial Hospital, Kaohsiung, Taiwan.; 18 Department of Pediatrics, China Medical University Hsinchu Hospital, Taiwan.; 19 College of Medicine, Fu-Jen Catholic University, Taipei, Taiwan.; 20 Department of Infant and Child Care, National Taipei University of Nursing and Health Sciences, Taipei, Taiwan.

**Keywords:** epigenotype, MassARRAY, Netchine-Harbison clinical scoring system, phenotype, quantitative DNA methylation, Silver-Russell syndrome

## Abstract

**Background:** Silver-Russell syndrome (SRS; OMIM #180860) is a clinically and genetically heterogeneous imprinting disorder characterized by prenatal and postnatal growth failure. The aim of this study was to identify the epigenotype-phenotype correlations in these patients using quantitative DNA methylation analysis.

**Methods:** One hundred and eighty-three subjects clinically suspected of having SRS were referred for diagnostic testing by the methylation profiling of *H19*-associated imprinting center (IC) 1 and imprinted *PEG1/MEST* regions using methylation-specific high-resolution melting analysis and methylation quantification with the MassARRAY assay. Correlations between quantitative DNA methylation status and clinical manifestations of the subjects according to the Netchine-Harbison (N-H) clinical scoring system for SRS were analyzed.

**Results:** Among the 183 subjects, 90 had a clinical diagnosis of SRS [N-H score ≥ 4 (maximum = 6)] and 93 had an SRS score < 4. Molecular lesions were detected in 41% (37/90) of the subjects with a clinical diagnosis of SRS, compared with 3% (3/93) of those with an N-H score < 4. The IC1 methylation level was negatively correlated with the N-H score. The molecular diagnosis rate was positively correlated with the N-H score. Thirty-one subjects had IC1 hypomethylation (IC1 methylation level <35% by the MassARRAY assay), seven had maternal uniparental disomy 7, and two had pathogenic copy number variants. Among the 90 subjects with an N-H score ≥ 4, the IC1 methylation level was significantly different between those with or without some clinical SRS features, including birth length ≤ 10th centile, relative macrocephaly at birth, normal cognitive development, body asymmetry, clinodactyly of the fifth finger, and genital abnormalities.

**Conclusions:** This study confirmed the suitability of the N-H clinical scoring system as clinical diagnostic criteria for SRS. Quantitative DNA methylation analysis using the MassARRAY assay can improve the detection of epigenotype‐phenotype correlations, further promoting better genetic counseling and multidisciplinary management for these patients.

## 1. Introduction

Silver-Russell syndrome (SRS; OMIM #180860) is a clinically and genetically heterogeneous imprinting disorder characterized by prenatal and postnatal growth failure. It was first reported independently by Silver et al. 1 in 1953 and Russell et al. 2 in 1954. Silver et al. 1 presented two children with short stature and hemihypertrophy, and Russell et al. 2 described five similar cases with intrauterine dwarfism, craniofacial dysostosis, and disproportionately short arms. SRS is associated with a constellation of clinical manifestations including intrauterine growth retardation without postnatal catch-up, relative macrocephaly, characteristic facial features, body and/or limb asymmetry, and fifth finger clinodactyly. The incidence of SRS has been estimated to range from 1:30,000 to 1:100,000, which may be underestimated because of the diverse and variable clinical manifestations. Most SRS patients are sporadic, although familial patients have occasionally been described 3,4. The relatively non-specific features of SRS make the clinical diagnosis difficult. An international consensus statement recommends using the Netchine-Harbison (N-H) clinical scoring system as clinical diagnostic criteria for SRS due to its high sensitivity and negative predictive values 4.

The common underlying mechanisms of SRS are hypomethylation of imprinting center 1 (IC1) on the paternal allele of the chromosome 11p15 region that regulates the *IGF2/H19* locus (seen in 30-60% of patients), and maternal uniparental disomy of chromosome 7 (mUPD7) (seen in 5-10% of patients). Rare cytogenetic rearrangements have also been described in 1% to 2% of cases. Gain of methylation of IC2, copy number variations, and sequence variants in *CDKN1C, HMGA2, IGF2*, or *PLAG1* were recently identified as (epi)genetic alterations associated with SRS. However, the molecular etiology remains unknown in a substantial proportion of SRS patients 3-12.

Molecular analysis can help to categorize subjects with SRS into subgroups, which can provide additional information on the natural course and for genetic counseling. Since pre- and postnatal growth retardation are relatively common and non-specific conditions probably caused by genetic, maternal or other environmental factors, complete phenotypic records and timely molecular analysis for SRS are important. Phenotype and genotype/epigenotype correlations in SRS patients have been described in the literature 6,7,9,11,13-16. SRS patients with IC1 hypomethylation are more likely to present with “classical” SRS and the more common characteristics of asymmetry, fifth finger clinodactyly and congenital anomalies compared with patients with mUPD7 9.

The MassARRAY assay is an accurate, sensitive, and reliable technique for cost‐effective and high‐throughput methylation analysis which can promote the detection of disease genes and increase our understanding of epigenetic modifications 17. Presently, only a few reports have analyzed quantitative DNA methylation and investigated the epigenotype‐phenotype correlations in subjects with SRS 18,19. The aim of this study was to characterize epigenotype‐phenotype relationships in Taiwanese patients with SRS according to the N-H clinical scoring system using quantitative DNA methylation analysis with the MassARRAY assay.

## 2. Patients and Methods

### 2.1. Study Population

We conducted a retrospective chart review of 183 subjects with clinically suspected SRS (89 males and 94 females; age range, 0 days to 36 years) who were referred for diagnostic testing from January 2013 through December 2022 at MacKay Memorial Hospital, Taipei, Taiwan. The cohort in the present study (n=183) is part of that of our previous study (n=206) 11. The number of patients less in the second study was because the quantitative DNA methylation analysis with the MassARRAY assay did not perform for the other 23 patients. This subset also qualifies for N-H score based on the collected information. All information was collected from the medical records. The chart review was performed by a single author (HYL) to ensure constant extraction of information. Written informed consent was obtained from a parent if the patient was a child and from the patient if they were over 18 years of age. The study was approved by the Ethics Committee of MacKay Memorial Hospital, Taipei, Taiwan.

### 2.2. Clinical Assessments

Clinical manifestations were recorded and analyzed according to the diagnostic criteria of the N-H clinical scoring system 4 which includes the following six scoring items (subjects with a score ≥ 4 points were classified as having SRS): (1) small for gestational age [birth weight and/or birth length ≤ 2 standard deviation score (*z* score)]; (2) postnatal growth failure (height at 24 ±1 months ≤ -2 *z* score or height ≤ -2 *z* score below the midparental target height); (3) relative macrocephaly at birth (head circumference at birth ≥ 1.5 *z* score above the birth weight and/or length *z* score); (4) frontal bossing forehead projecting beyond the facial plane on a side view as a toddler (1-3 years)]; (5) body asymmetry [leg length discrepancy ≥ 0.5 cm, arm asymmetry, or leg length discrepancy < 0.5 cm with at least two other asymmetrical body parts (one non-face)]; and (6) feeding difficulties and/or low body mass index (≤ -2 *z* score at 24 months) or a history of feeding tube use in infancy.

Other data obtained from the records included sex, age at diagnosis, history of conception by assisted reproductive technology (ART), as well as birth length, weight, and head circumference *z* scores. *Z* scores for height, weight, and head circumference were computed using standard growth tables for the Taiwanese population 20. A *z* score was derived by subtracting the population mean from each subject's raw score, and then dividing the difference by the standard deviation of the population.

### 2.3. Molecular Studies

#### DNA Extraction and Bisulfite Treatment

All DNA was extracted from peripheral blood using a Chemagic DNA Blood Kit (Chemagen, Baesweiler, Germany) and a MethylCode Bisulfite Conversion Kit (Invitrogen, Carlsbad, CA) according to the manufacturers' instructions. All diagnostic examinations were carried out by profiling the methylation of *H19*-associated IC1 and the imprinted *PEG1/MEST* regions using methylation-specific high-resolution melting (MS-HRM) analysis with a methylation-specific polymerase chain reaction (PCR) assay and the MassARRAY assay. mUPD7 was tested by analyzing the methylation status of the imprinted *PEG1/MEST* region using MS-HRM analysis and the MassARRAY assay. Hypermethylation of *PEG1/MEST* region was the first-line screening tool. Positive cases were subsequently confirmed mUPD7 by haplotype analysis using single nucleotide polymorphism genotyping or short tandem repeat markers. In addition, we used a whole genome strategy to detect copy number changes and loss of heterozygosity. The detailed procedures have been described previously 11,21-23.

### 2.4. Methylation Analysis Using Methylation-Sensitive High-Resolution Melting

For HRM analysis, bisulfite-treated DNA was analyzed using a BIO-RAD CFX Connect™ Real Time System (Bio-Rad Laboratories, CA, USA). The detailed procedures have been described previously 23. Supplementary Table 1 shows the primers for MS-HRM. All of the steps included positive and negative controls along with the patients' samples.

### 2.5. Methylation Analysis Using the MassARRAY EpiTYPER Platform

Amplification of bisulfite-treated DNA with *H19*-associated IC1 and the imprinted *PEG1/MEST* region was performed*.* The quantitation of DNA methylation was carried out using the MassARRAY EpiTYPER platform (Sequenom, San Diego, CA, USA) as previously described 17,23,24. Supplementary Table 2 shows the primer sequences for the bisulfite PCR.

All diagnostic examinations were performed by methylation profiling of *H19*-associated IC1 and the imprinted *PEG1/MEST* region using MS-HRM and high-resolution quantitative methylation profiling with a methylation-specific PCR assay using the MassARRAY EpiTYPER platform (Sequenom, San Diego, CA, USA). DNA samples from 100 age-matched healthy controls were included in this study to set up the MassARRAY methylation panel and define the normal range of methylation levels. The reference ranges of IC1 and *MEST* methylation levels were determined as the mean plus two standard deviations.

### 2.6. Data and Statistical Analysis

For the subjects with a clinical diagnosis of SRS (N-H score ≥ 4), we compared the IC1 methylation level between those with or without certain clinical SRS features, as well as those with or without each N-H clinical SRS feature. The N-H clinical features of subjects with IC1 hypomethylation versus those with mUPD7 were also analyzed using the Student's *t*-test for continuous variables and Fisher's exact test for categorical variables. Two-tailed *p*-values were calculated. The relationships between N-H score and IC1 methylation level as well as molecular diagnosis rate of the 183 subjects were computed using Pearson's correlation coefficient (*r*), and testing for statistical significance (*p* < 0.05) was performed using Fisher's *r-z* transformations. Relationships between IC1 methylation level and *z* scores of birth height, weight, and head circumference of the subjects with IC1 hypomethylation were also analyzed. All statistical analyses were conducted using SPSS version 11.5 (SPSS Inc., Chicago, Illinois, USA). Statistical significance was set at *p* < 0.05.

## 3. Results

**Figure 1:**
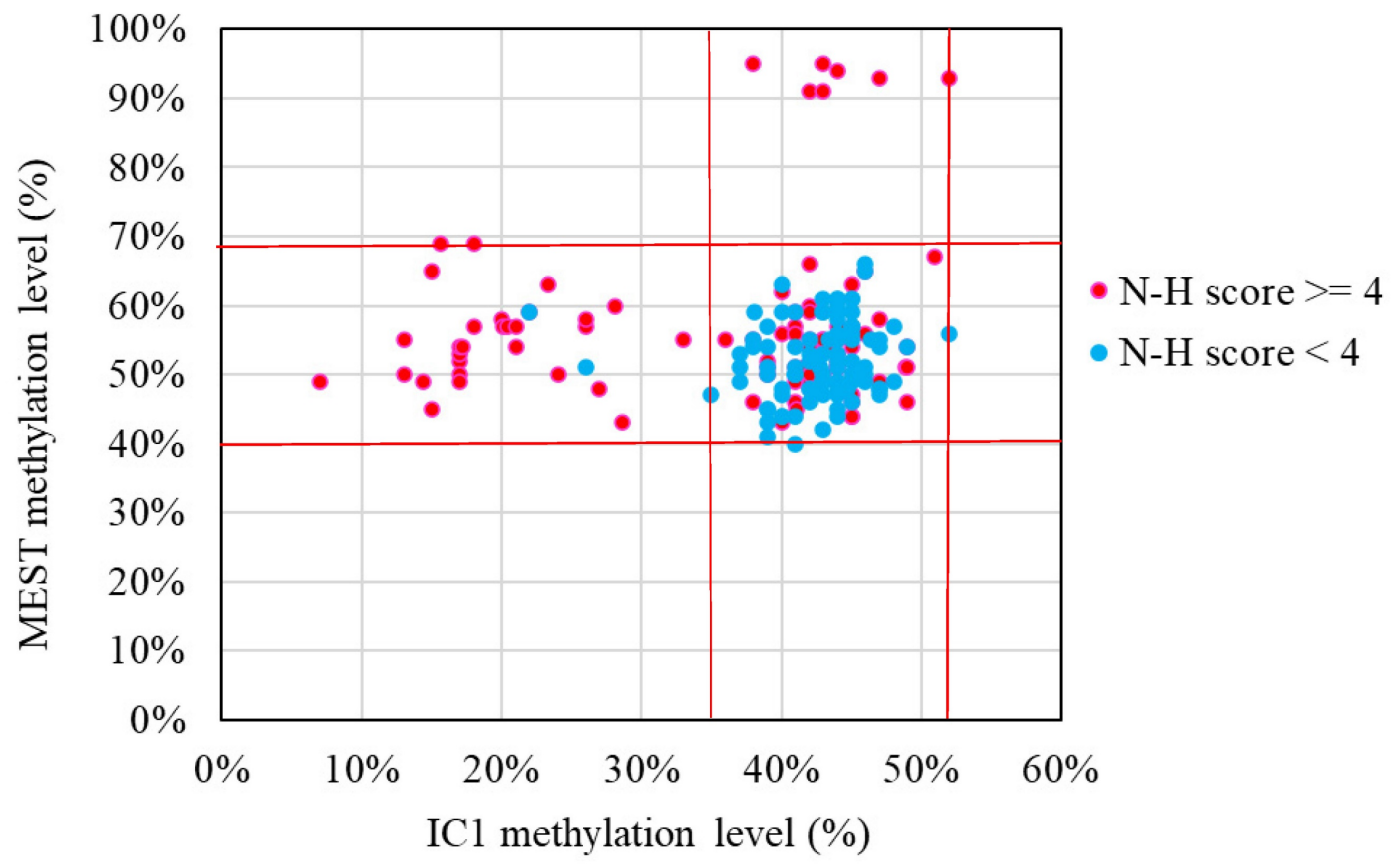
IC1 and *MEST* methylation levels in the 183 subjects clinically suspected of having Silver-Russell syndrome (SRS) in this study. Subjects with hypermethylated *MEST* data represented maternal uniparental disomy of chromosome 7. N-H score, Netchine-Harbison clinical scoring system; IC, imprinting center. *Red lines represent upper and lower limits of the reference ranges by the MassARRAY assay (IC1 methylation level: 35-52%, *MEST* methylation level: 40-69%).

**Figure 2:**
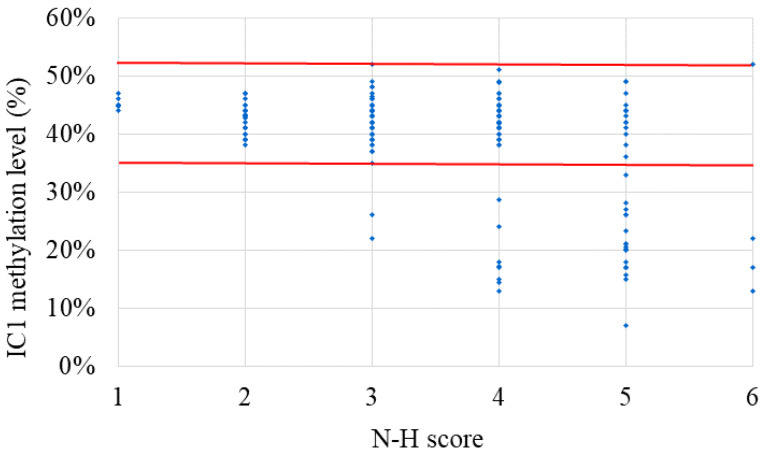
The IC1 methylation level in the 183 subjects clinically suspected of having Silver-Russell syndrome (SRS) subjects with each N-H score group (N-H score = 1 to 6). N-H score, Netchine-Harbison clinical scoring system; IC, imprinting center. *Red lines represent upper and lower limits of the reference range of IC1 methylation level (35-52%) by the MassARRAY assay. The IC1 methylation level was negatively correlated with the N-H score (n = 183, *r* = -0.451, *p* < 0.01).

**Figure 3:**
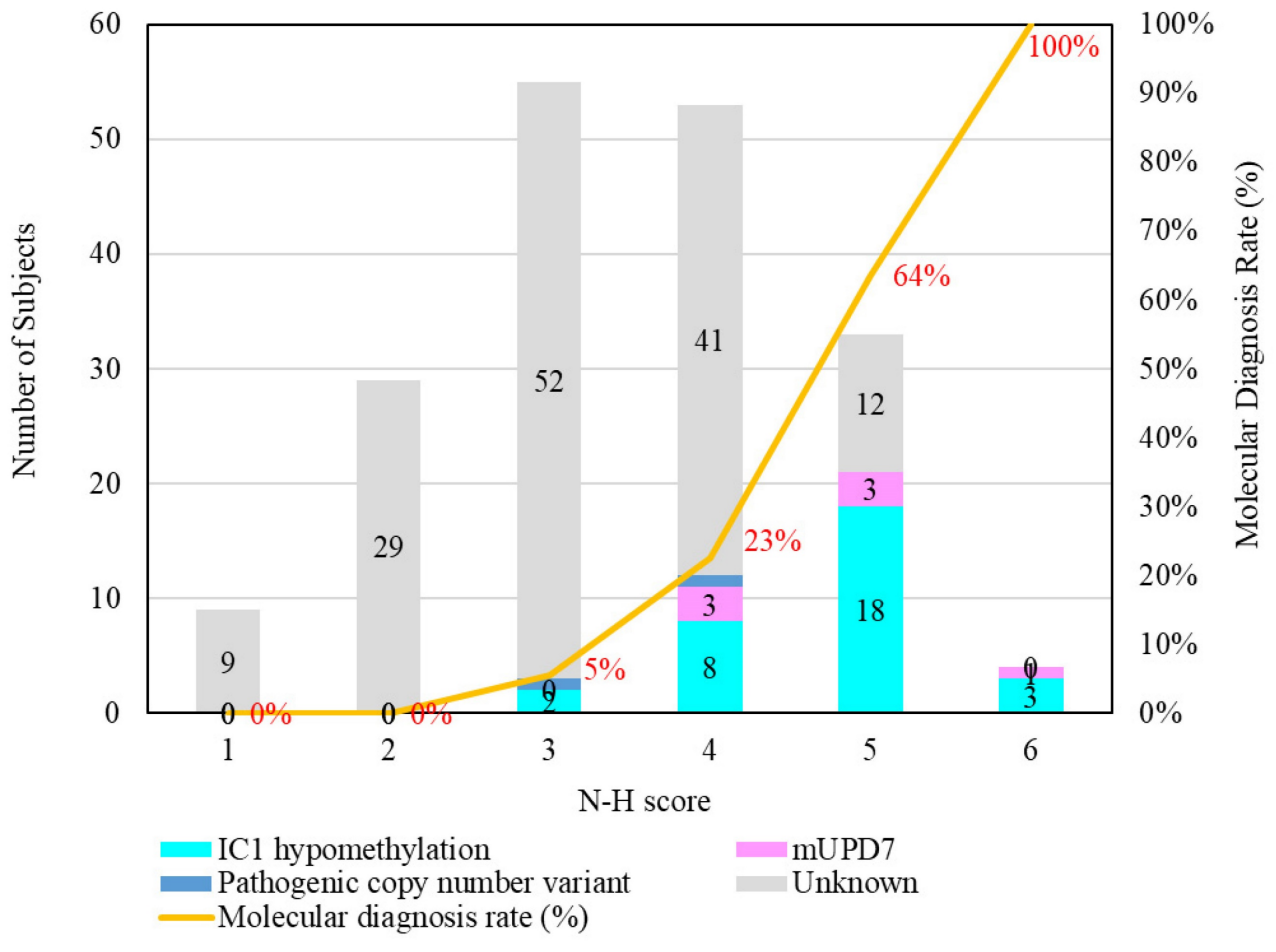
Number of subjects with different molecular defects and each N-H score group. The molecular diagnosis rate was positively correlated with the N-H score (n = 183, *r* = 0.542, *p* < 0.01). The molecular diagnosis rate for each N-H score group was as follows: 100% (N-H score = 6, n = 4), 64% (N-H score = 5, n = 33), 23% (N-H score = 4, n = 53), and 3% (N-H score < 4, n = 93). mUPD7, maternal uniparental disomy of chromosome 7; IC, imprinting center.

**Figure 4:**
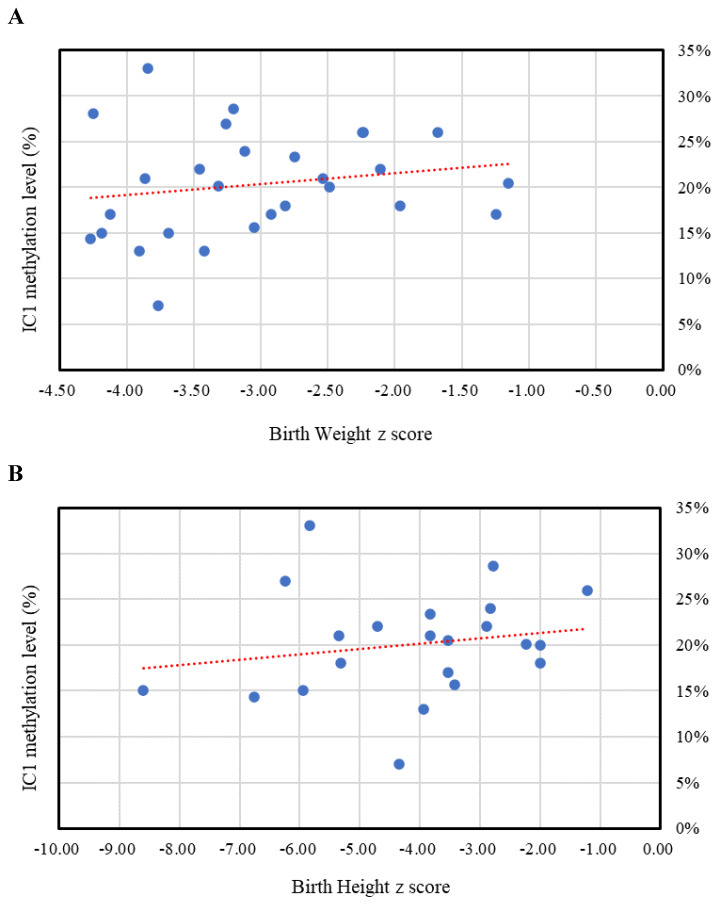

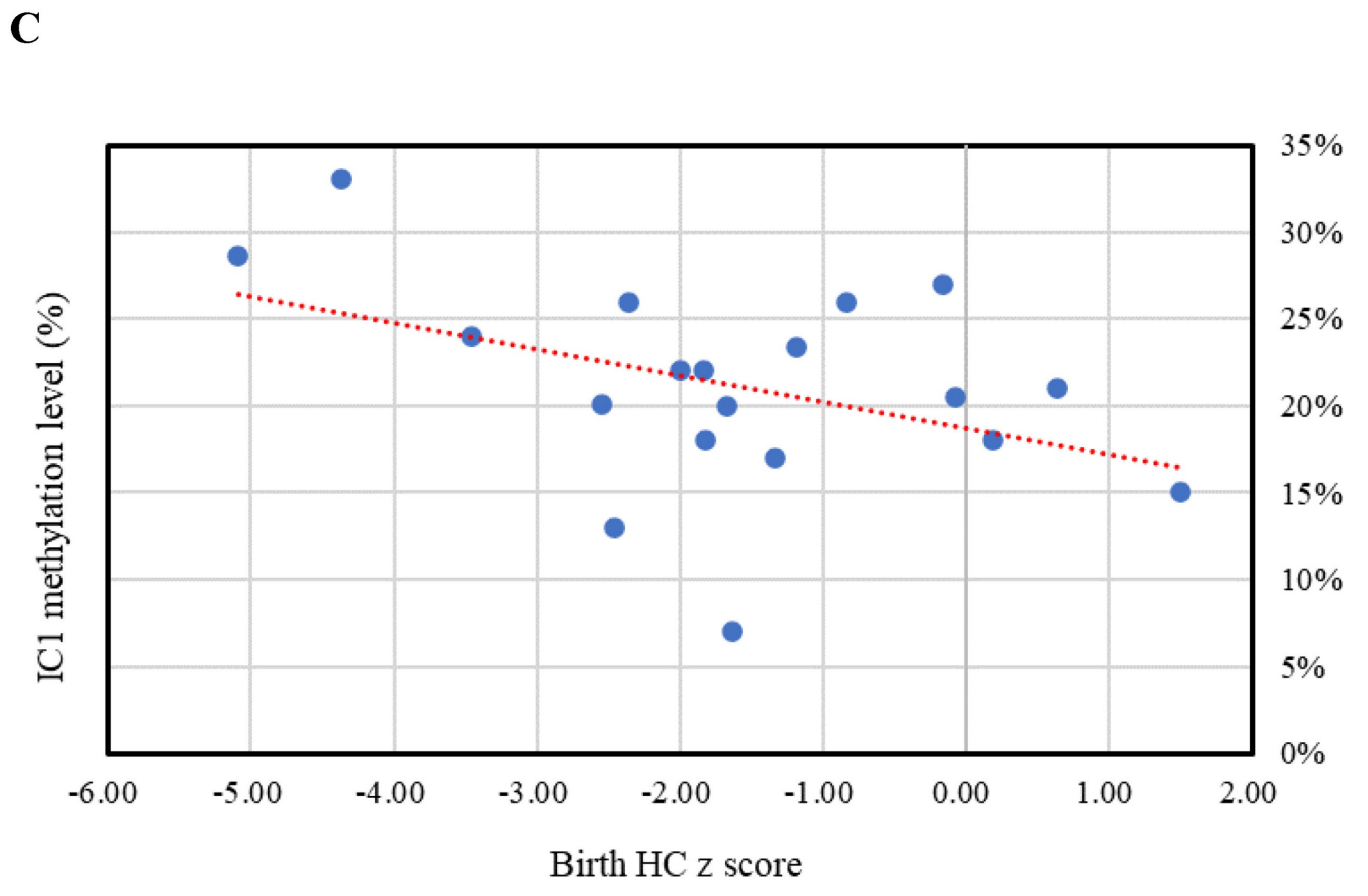
The relationships between IC1 methylation level by the MassARRAY assay and *z* scores of birth weight, birth height, and birth head circumference (HC) in the Silver-Russell syndrome subjects with IC1 hypomethylation. (A) Birth weight *z* score (n = 28, *r* = 0.184, *p* > 0.05). (B) Birth height *z* score (n = 22, *r* = 0.184, *p* > 0.05). (C) Birth HC *z* score (n = 19, *r* = -0.416, *p* > 0.05). IC, imprinting center.

The results of the MassARRAY quantification were in line with the results of the MS-HRM, which we consider a qualitative method. Among the 183 subjects, 31 had IC1 hypomethylation, seven had mUPD7, and two had pathogenic copy number variants (Table 1). The whole genome arrays identified that one subject had a microdeletion on chromosome 22q11.21q11.22 (1.497Mb deletion) with an N-H score of 3, and one subject had a microduplication on chromosome 7p14.1 (379Kb duplication) with an N-H score of 4. Figure 1 shows the IC1 and *MEST* methylation levels in the 183 subjects, of whom 90 were classified as having a clinical diagnosis of SRS [N-H score ≥ 4 (maximum = 6)], and 93 had an N-H score < 4. The IC1 methylation level was negatively correlated with the N-H score (n = 183, *r* = -0.451, *p* < 0.01). The mean IC1 methylation level by the MassARRAY assay for each N-H score group was as follows: 26% (N-H score = 6, n = 4), 31% (N-H score = 5, n = 33), 39% (N-H score = 4, n = 53), and 42% (N-H score < 4, n = 93) (Figure 2). The molecular diagnosis rate was positively correlated with the N-H score (n = 183, *r* = 0.542, *p* < 0.01). The molecular diagnosis rate for each N-H score group was as follows: 100% (N-H score = 6, n = 4), 64% (N-H score = 5, n = 33), 23% (N-H score = 4, n = 53), and 3% (N-H score < 4, n = 93) (Figure 3). Molecular lesions were detected in 41% (37/90) of the subjects with a clinical diagnosis of SRS (N-H score ≥ 4), compared to 3% (3/93) of those with an N-H score < 4. Among the 90 subjects with an N-H score ≥ 4, the IC1 methylation level was found to be significantly different (*p* < 0.05) between those with or without some clinical SRS features, including birth length ≤ 10th centile (IC1 methylation level: 34% vs 43%), relative macrocephaly at birth (31% vs 41%), normal cognitive development (34% vs 40%), asymmetry (face/body/limbs) (33% vs 39%), clinodactyly of the fifth finger (32% vs 39%), and genital abnormalities (e.g. cryptorchidism, hypospadias) (29% vs 37%) (Table 2). Among the six items of the N-H clinical scoring system, the IC1 methylation level was found to be significantly lower in the subjects with the item “body asymmetry” than in those without this item (*p* < 0.05) (Table 3). Table 4 shows the clinical features and IC1 and *MEST* methylation levels of the 31 subjects with IC1 hypomethylation and seven subjects with mUPD7. The mean age at diagnosis was 4.4 years in those with IC1 hypomethylation, and 7.8 years in those with mUPD7. Of the subjects with IC1 hypomethylation, 74% had body asymmetry, compared with 57% of those with mUPD7. Among the 31 SRS subjects with IC1 hypomethylation, there seemed to be trends between a lower IC1 methylation level and lower birth height, lower birth weight, and larger birth head circumference, although the *P* values did not reach statistical significance (*p* > 0.05) (Table 5 and Figure 4). Among the 90 subjects with a clinical diagnosis of SRS (N-H score ≥ 4), five (5.5%) were conceived by ART. Two of these subjects were identified to have IC1 hypomethylation, with N-H scores of 5 (IC1 methylation level of 17%) and 4 (IC1 methylation level of 15%), respectively. The other three subjects had normal molecular study results, and their N-H scores were 5, 5 and 4, respectively.

## 4. Discussion

To the best of our knowledge, this is the first cohort study to analyze quantitative DNA methylation using the MassARRAY assay and investigate the epigenotype‐phenotype correlations in clinically diagnosed SRS subjects according to the N-H clinical scoring system in Taiwan. We used the MassARRAY assay to analyze methylation levels at *H19*-associated IC1 and imprinted *PEG1/MEST* regions, and found that a lower IC1 methylation level tended to be associated with higher N-H score and greater disease severity in clinically suspected SRS subjects.

Among the 90 SRS subjects with an N-H score ≥ 4, a lower IC1 methylation level was associated with lower birth length, relative macrocephaly at birth, normal cognitive development, asymmetry (face/body/limbs), clinodactyly of the fifth finger, and genital abnormalities (e.g. cryptorchidism, hypospadias). In addition, 53 (59%) of these 90 subjects had unknown epigenetic or genetic defects, suggesting that a group of molecular assays are necessary to define the epigenotype‐phenotype correlations.

The suitability of the N-H clinical scoring system developed by Wakeling et al. 4 as clinical diagnostic criteria for SRS has been confirmed in recent studies 10,12. In the present study, the molecular diagnosis rate was positively correlated with the N-H score (*p* < 0.01). Our results demonstrated the feasibility of using the N-H clinical scoring system to predict outcomes of molecular abnormalities. Wakeling et al. 4 recommended the threshold for SRS molecular testing was ≥3 of six criteria, which was lower than that needed for a clinical diagnosis of SRS (≥4 of six criteria). Consistently, two subjects with an N-H score of 3 were detected to have IC1 hypomethylation in our cohort.

A number of studies have described the clinical and molecular findings for Western European patients with SRS 5-7,9,13-16; however, only a few studies have been performed in Asian patients 3,10,11,19. SRS is primarily a clinical diagnosis; however, molecular analysis helps to confirm the clinical diagnosis and classify the subtype 4. In our cohort of 90 subjects with a clinical diagnosis of SRS (N-H score ≥ 4), the overall molecular defect detection rate was 41%. The frequencies of the different molecular defects of IC1 hypomethylation, mUPD7, and pathogenic copy number variants were 32%, 8% and 1%, respectively, which are in agreement with those reported in the literature 6,8,13. Using the MassARRAY assay, we confirmed the molecular defects of SRS in 40% of 90 subjects with an N-H score ≥ 4. The molecular diagnosis rates in this study were consistent with those reported by Fuke et al. 12. This indicates that the MassARRAY assay is a reliable test to confirm clinically suspected SRS.

In this study, we used the MassARRAY EpiTYPER mass spectrometer analysis technology platform. Accurate analysis of methylation at the imprinting control regions of *H19*-associated IC1 and *PEG1/MEST* is an important tool for the molecular diagnosis of SRS. The MassARRAY assay can more accurately analyze methylation variations of nucleic acids compared with the lower accuracy of qualitative (methylation‐specific PCR) and semi‐quantitative (southern blotting and methylation‐sensitive multiplex ligation probe analysis) methods 23,24. In addition, analyzing the methylation status of the imprinted *PEG1/MEST* region is a cost-effective screening method for mUPD7 molecular diagnosis prior to microsatellite analysis of the parents' DNA, which can help to increase the diagnostic rate 25. In this study we also used MS-HRM, which has been reported to be a sensitive, rapid, and cost‐effective method for screening methylation changes at the *H19*-associated IC1 region in SRS 22,26.

Netchine et al. 6 reported that birth weight and length were significantly lower, and a prominent forehead, body asymmetry, and relative macrocephaly were significantly more frequent in SRS patients with IC1 hypomethylation than in those without IC1 hypomethylation. In addition, Lee et al. 18 reported that IC1 methylation scores quantified by methylation‐specific pyrosequencing were positively correlated with the birth height and weight of their patients with SRS (n = 20) and Beckwith-Wiedemann syndrome (n = 18). Furthermore, Fuke et al. 19 reported that the methylation index of *H19* differentially methylated regions by pyrosequencing analysis was positively correlated with birth length and weight in 43 Japanese SRS patients with IC1 hypomethylation. In the present study, we further demonstrated the relationship between IC1 methylation level using the MassARRAY assay and the existence of certain SRS features quantitatively.

Fuke et al. 19 analyzed the epigenotype/phenotype in their cohort, and reported that patients with IC1 hypomethylation had a greater reduction in birth length and weight, more preserved birth occipitofrontal circumference, and more frequent body asymmetry than patients with mUPD7. Wakeling et al. 4 reported that asymmetry, fifth finger clinodactyly and congenital anomalies were more commonly seen in subjects with IC1 hypomethylation, whereas learning difficulties and referral for speech therapy were more common in subjects with mUPD7. In the present study, the mean age at diagnosis of the subjects with IC1 hypomethylation was earlier than in those with mUPD7 (4.4 years versus 7.8 years), suggesting that the former type may be associated with more significant SRS clinical manifestations than the latter type. In addition, body asymmetry was more frequently seen in the subjects with IC1 hypomethylation than in those with mUPD7 (74% versus 57%). Our results are consistent with those of the previous studies.

Lin et al. 21 reported that a whole genome approach could provide information on the etiology of SRS, and that if no epimutations are identified in patients with typical SRS, microdeletions should be suspected. We identified two patients with pathogenic copy number variants in our cohort, and they had N-H scores of 3 and 4, respectively. Since many microdeletion and microduplication syndromes present with growth retardation and dysmorphism, which overlap with SRS, health care professionals should keep in mind the possibility of pathogenic copy number variants when no epimutations are detected in patients with SRS 21.

ART-conceived embryos have been reported to be associated with a higher incidence of various imprinting disorders such as SRS, Beckwith-Wiedemann syndrome, Prader-Willi syndrome, and Angelman syndrome due to genetic and epigenetic variations during embryonic development. Different phases of ART, such as ovarian stimulation, *in vitro* fertilization, intracytoplasmic sperm injection, and the culture medium of the fertilized egg may affect the most important period of epigenetic reprogramming 27. In our cohort, five SRS subjects (5/90, 5.5%) were conceived by ART, and IC1 hypomethylation was detected in two of them. Wakeling et al. 9 reported that children conceived by ART seemed to have a higher incidence of IC1 hypomethylation compared with the general population. In addition, Hattori et al. 28 conducted a nationwide epidemiological study in Japan, and reported that 11.9% (8/67) of their ART subjects had SRS, representing an 8.91-fold increased frequency of SRS associated with ART. Our results are similar to the previous reports.

### Limitations

As a retrospective study, not all clinical data were available for all of our subjects. Because of the limitation of the study design, none of the monogenic causes of SRS were analyzed in this study. MassARRAY was not performed for additional loci, including chromosome 14q32 (Temple syndrome), mUPD20, mUPD16, and multi-locus imprinting disturbance (MLID) 4. In addition, the age range of our subjects was quite wide, as was the degree of disease severity. The relatively small sample size of the different molecular etiologies reflects the rare nature of this genetic disease. Therefore, larger cohort studies with longer follow-up are needed to clarify our findings.

## 5. Conclusions

This 10-year review is the first cohort study to analyze quantitative DNA methylation using the MassARRAY assay and investigate epigenotype‐phenotype correlations in clinically diagnosed SRS subjects according to the N-H clinical scoring system in Taiwan. Our findings confirmed the suitability of the N-H clinical scoring system as clinical diagnostic criteria for SRS. The N-H score was positively correlated with the molecular diagnosis rate. A lower IC1 methylation level was associated with a higher N-H score and greater disease severity of the SRS subjects. Quantitative DNA methylation analysis using the MassARRAY assay can improve the detection of epigenotype‐phenotype correlations, further promoting better genetic counseling and multidisciplinary management for these patients.

## Supplementary Material

Supplementary tables.

## Figures and Tables

**Table 1:** Epigenetic and genetic defects and molecular diagnosis rates of the 183 subjects clinically suspected of having Silver-Russell syndrome according to the Netchine-Harbison (N-H) clinical scoring system.

N-H score (Maximum = 6)	N	IC1 methylation level (%)	IC1 hypomethylation	mUPD7	Pathogenic copy number variant	Unknown	Molecular diagnosis	Molecular diagnosis rate (%)
6	4	26%	3	1	0	0	4	100%
5	33	31%	18	3	0	12	21	64%
4	53	39%	8	3	1	41	12	23%
3	55	42%	2	0	1	52	3	5%
2	29	43%	0	0	0	29	0	0%
1	9	45%	0	0	0	9	0	0%

IC, imprinting center; mUPD7, maternal uniparental disomy of chromosome 7.

**Table 2:** Quantitative IC1 methylation level using the MassARRAY assay in the 90 subjects with a clinical diagnosis of SRS with or without each SRS feature.

Clinical SRS features	With or without certain features	N	*IC1 methylation level (mean ± standard deviation) (%)	*p* value
Birth weight ≤ 10th centile	Without	11	40 ± 7	0.224
	With	79	35 ± 12	
Birth length ≤ 10th centile	Without	13	43 ± 4	**0.014**
	With	77	34 ± 12	
Relative macrocephaly at birth	Without	43	41 ± 6	**<0.001**
	With	47	31 ± 13	
No catch-up growth; height ≤ 3rd centile	Without	7	37 ± 12	0.801
	With	83	36 ± 12	
Normal head circumference; OFC ≥ 3rd centile and ≤ 97th centile	Without	24	39 ± 9	0.086
	With	66	34 ± 12	
Normal cognitive development	Without	28	40 ± 9	**0.024**
	With	62	34 ± 12	
Asymmetry (face/body/limbs)	Without	42	39 ± 10	**0.007**
	With	48	33 ± 13	
Triangular shaped face	Without	19	40 ± 8	0.068
	With	71	35 ± 12	
High/bossing forehead	Without	12	39 ± 11	0.231
	With	78	35 ± 12	
Others: eg, small chin, thin lips, down turned corners of the mouth, late closure of fontanelle	Without	32	37 ± 10	0.393
	With	58	35 ± 13	
Clinodactyly of the fifth finger	Without	45	39 ± 10	**0.002**
	With	45	32 ± 13	
Genital abnormalities (eg, cryptorchidism, hypospadias)	Without	80	37 ± 11	**0.046**
	With	10	29 ± 13	
Others: eg, brachymesophalangy, syndactyly toes, inguinal hernia, pigmentary changes	Without	63	37 ± 11	0.125
	With	27	33 ± 14	

IC, imprinting center; SRS, Silver-Russell syndrome; OFC, occipitofrontal circumference. The *p* value < 0.05 is presented in boldface. *Reference range: 35-52%.

**Table 3:** Quantitative IC1 methylation level using the MassARRAY assay in the 90 subjects with a clinical diagnosis of SRS in this study with or without each SRS feature according to the Netchine-Harbison (N-H) clinical scoring system.

Clinical SRS features	With or without certain features	N	*IC1 methylation level (mean ± standard deviation) (%)	*p* value
N-H score (1): SGA	Without	6	41 ± 5	0.240
	With	84	35 ± 12	
N-H score (2): Postnatal growth failure	Without	7	37 ± 12	0.801
	With	83	36 ± 12	
N-H score (3): Relative macrocephaly at birth	Without	24	39 ± 9	0.086
	With	66	34 ± 12	
N-H score (4): Protruding forehead	Without	12	39 ± 11	0.231
	With	78	35 ± 12	
N-H score (5): Body asymmetry	Without	42	39 ± 10	**0.007**
	With	48	33 ± 13	
N-H score (6): Feeding difficulties and/or low BMI	Without	48	34 ± 12	0.124
	With	42	38 ± 11	

*Reference range: 35-52%. SRS, Silver-Russell syndrome; IC, imprinting center; SGA, small for gestational age; BMI, body mass index. The *p* value < 0.05 is presented in boldface.

**Table 4:** Clinical features according to the Netchine-Harbison (N-H) clinical scoring system for the 31 SRS subjects with IC1 hypomethylation and seven subjects with mUPD7.

Clinical features	IC1 hypomethylation (n=31)	mUPD7 (n=7)	*p* value
Age at diagnosis (years)	4.4 ± 4.4	7.8 ± 8.3	0.123
N-H score (1): SGA	31 (100%)	7 (100%)	ND
N-H score (2): Postnatal growth failure	28 (90%)	7 (100%)	0.405
N-H score (3): Relative macrocephaly at birth	27 (87%)	5 (71%)	0.318
N-H score (4): Protruding forehead	26 (84%)	6 (86%)	0.907
N-H score (5): Body asymmetry	23 (74%)	4 (57%)	0.383
N-H score (6): Feeding difficulties and/or low BMI	11 (35%)	4 (57%)	0.302
Total N-H score (maximum = 6)	4.7 ± 7.4	4.7 ± 7.6	0.988
IC1 methylation level (%)*	20 ± 6	44 ± 4	**<0.001**
*MEST* methylation level (%)**	55 ± 6	93 ± 2	**<0.001**

Reference ranges by the MassARRAY assay: *35-52%; **40-69%. SRS, Silver-Russell syndrome; IC, imprinting center; mUPD7, maternal uniparental disomy of chromosome 7; SGA, small for gestational age; BMI, body mass index; ND, not defined. The *p* value < 0.05 is presented in boldface.

**Table 5:** Gender, *z* scores of birth weight, height, and head circumference, Netchine-Harbison (N-H) score, and IC1 and *MEST* methylation levels by the MassARRAY assay for the 31 SRS subjects with IC1 hypomethylation.

No.	Gender	Birth weight *z* score	Birth height* z* score	Birth HC *z* score	N-H score (Maximum = 6)	IC1 methylation level (%)*	*MEST* methylation level (%)**
1	M	-3.42	NA	NA	6	13%	55%
2	M	-4.12	NA	NA	6	17%	52%
3	M	-3.46	-4.71	-1.83	6	22%	59%
4	F	-3.76	-4.35	-1.64	5	7%	49%
5	M	-4.18	-8.60	NA	5	15%	65%
6	M	-3.05	-3.43	NA	5	16%	69%
7	F	NA	NA	NA	5	17%	50%
8	M	-2.92	-3.53	-1.33	5	17%	53%
9	M	NA	NA	NA	5	17%	49%
10	F	-1.96	-5.32	0.18	5	18%	69%
11	M	-2.49	-2.00	-1.67	5	20%	58%
12	M	-3.32	-2.24	-2.55	5	20%	57%
13	M	-1.15	-3.53	-0.08	5	20%	57%
14	F	-3.86	-5.35	NA	5	21%	57%
15	M	-2.54	-3.82	0.64	5	21%	54%
16	M	-2.74	-3.82	-1.18	5	23%	63%
17	F	-2.24	NA	NA	5	26%	57%
18	M	-2.23	NA	-0.83	5	26%	58%
19	M	-3.26	-6.24	-0.17	5	27%	48%
20	F	-4.25	NA	NA	5	28%	60%
21	F	-3.84	-5.84	-4.36	5	33%	55%
22	F	-3.90	-3.95	-2.45	4	13%	50%
23	M	-4.27	-6.76	NA	4	14%	49%
24	M	-3.69	-5.94	1.50	4	15%	45%
25	F	-1.24	NA	NA	4	17%	54%
26	M	NA	NA	NA	4	17%	54%
27	F	-2.82	-2.00	-1.82	4	18%	57%
28	F	-3.12	-2.82	-3.45	4	24%	50%
29	M	-3.20	-2.79	-5.09	4	29%	43%
30	F	-2.11	-2.89	-2.00	3	22%	59%
31	M	-1.68	-1.21	-2.36	3	26%	51%

Reference ranges: *35-52%; **40-69%. SRS, Silver-Russell syndrome; HC, head circumference; IC, imprinting center; NA, not available.

## Abbreviations

SRS: Silver-Russell syndrome
N-H: Netchine-Harbison
IC: imprinting center
mUPD7: maternal uniparental disomy of chromosome 7
ART: assisted reproductive technology
MS-HRM: methylation-specific high-resolution melting
PCR: polymerase chain reaction
