# Supplementary material for: Quantitative DNA Methylation Analysis and Epigenotype-Phenotype Correlations in Taiwanese Patients with Silver-Russell Syndrome
Source: Int J Med Sci. 2024 Jan 1;21(1):8–18. doi: 10.7150/ijms.84154 (PMC10750329; doi:10.7150/ijms.84154)
Supplement: Supplementary file 1 — Supplementary tables. [file ijmsv21p0008s1.pdf]

Supplementary Table 1. Primers for MS-HRM.

Gene	Primer sequence	Amplicon size (bp)	Annealing temperature
H19	F: 5’- GGG AGA GTT TGT GAG GT<br>R: 5’- AAA TCC CCA CAA CCG CTA AAC	137	56℃
MEST (UPD7)	F: 5’- GTT GTT GGT TAG TTT TGT<br>R: 5’- ACC CCC TCC TCA AAT AAA	279	56℃

Supplementary Table 2. Primer sequences for bisulfite PCR.

Sequence Description	Tm	Sequence	Size	Number of CpG investigated
H19Amp10-10F	61℃	aggaagagagGAGATTTGAGGTGAATTTTAGGGA	337bp	20
H19Amp10-T7R		cagtaatacgactcactatagggagaaggctCAAAACAAAATCCCCACAACC		
MEST-Amp8-10F	58℃	aggaagagagTTTTAAAGGAGTTATTGTTAGAGGGG	598bp	66
MEST-Amp8-T7R		cagtaatacgactcactatagggagaaggctACCCCCTCCTCAAATAAACAAAA		
